# Reduced Fhit expression is associated with mismatch repair deficiency in human advanced colorectal carcinoma

**DOI:** 10.1038/sj.bjc.6600501

**Published:** 2002-08-12

**Authors:** H Andachi, K Yashima, M Koda, K Kawaguchi, A Kitamura, A Hosoda, Y Kishimoto, G Shiota, H Ito, M Makino, N Kaibara, H Kawasaki, Y Murawaki

**Affiliations:** Second Department of Internal Medicine, Faculty of Medicine, Tottori University, Yonago 683-8504, Japan; Department of Clinical Pharmacology, Faculty of Medicine, Tottori University, Yonago 683-8504, Japan; First Department of Pathology, Faculty of Medicine, Tottori University, Yonago 683-8504, Japan; First Department of Surgery, Faculty of Medicine, Tottori University, Yonago 683-8504, Japan

**Keywords:** colorectal carcinoma, immunohistochemistry, Fhit, Msh2, Mlh1, p53

## Abstract

The *Fragile Histidine Triad* gene, encompassing the FRA3B fragile site at chromosome 3p14.2, is a candidate tumour suppressor gene involved in multiple tumour types including colorectal carcinomas. Recently, it has been reported that the *Fragile Histidine Triad* gene may be a target of damage in a fraction of mismatch deficient tumours. To explore this hypothesis, we analysed both Fragile histidine triad and mismatch repair protein (Msh2 and Mlh1) expression using immumohistochemical methods in 52 advanced colorectal carcinomas (19 well-, 17 moderately-, and 16 poorly-differentiated). In addition, we examined whether the Fragile histidine triad and mismatch repair protein expression correlated with p53 expression and clinicopathological findings. Significant loss or reduction of Fragile histidine triad expression was noted in 18 of the 52 (34.6%) advanced colorectal carcinomas: 2 (10.5%) well-differentiated, 3 (17.6%) moderately-differentiated, 13 (81.3%) poorly-differentiated carcinomas, the frequency being significantly higher in the latter than that in the former two (*P*<0.0001). Loss of mismatch repair protein (mainly, Mlh1) expression was detected in 21 of the 52 (40.4%) colorectal carcinomas. Moreover, reduced Fragile histidine triad expression was significantly associated with absence of mismatch repair protein expression in the advanced colorectal carcinomas (*P*<0.0001). However, the Fragile histidine triad and mismatch repair protein expression was not significantly associated with p53 expression. These results suggested that reduced Fragile histidine triad expression might be correlated with mismatch repair expression, but not with p53 expression.

*British Journal of Cancer* (2002) **87**, 441–445. doi:10.1038/sj.bjc.6600501 www.bjcancer.com

© 2002 Cancer Research UK

## 

A candidate tumour suppressor gene, *Fragile Histidine Triad* (*FHIT*), was identified at chromosome 3p14.2 spanning the FRA3B common fragile site (Ohta *et al*, 1996). The *FHIT* gene consists of 10 exons, which distribute over a genomic region of >1 Mb. The Fragile histidine triad (Fhit) protein has been characterised as an Ap_3_A hydrolase molecule, which cleaves the Ap_4_A substrate that may be involved in the control of DNA replication and the cell cycle (Ohta *et al*, 1996). Frequent abnormal transcripts were found in a variety of human cancers including those of the digestive tract, lung, breast, and head and neck (Ohta *et al*, 1996; Croce *et al*, 1999; Kitamura *et al*, 2001). The majority of these abnormalities include aberrant mRNA transcripts, with the absence of one or more exons within the mRNA. Genomic analysis demonstrated frequent allelic loss and homozygous deletions (Ohta *et al*, 1996; Croce *et al*, 1999).

In CRCs, Ohta *et al* (1996) reported that aberrant transcripts of the *FHIT* gene were detected in three of eight tumours by nested RT–PCR. In contrast, Thiagalingam *et al* (1996) reported that aberrant transcripts were rare using RT–PCR, suggesting that *FHIT* is inactivated by an unusual mechanism or that it plays a role in relatively few colorectal tumours. Other reports suggested that Fhit does indeed play a role in the development and progression of colorectal carcinomas (CRCs) (Hibi *et al*, 1997; Hao *et al*, 2000; Luceri *et al*, 2000; Mori *et al*, 2001).

Defects in the DNA mismatch repair (MMR) system are involved in the carcinogenesis and tumour progression of sporadic and inherited human cancers (Eshleman *et al*, 1996; Kinzler and Vogelstein, 1996). MMR deficiency leads to the accumulation of base-base mismatches and short insertion/deletion mispairs, generated as a consequence of DNA replication errors and homologous recombinations. Inherited mutations of the *MLH1* and *MSH2* genes have been demonstrated as the cause of more than 90% of hereditary non-polyposis colorectal cancers (HNPCC) (Peltomaki and Vasen, 1997). MMR genes are also involved in the development of a subset of sporadic colorectal, gastric and endometrial tumours (Duggan *et al*, 1994; Halling *et al*, 1999; Ward *et al*, 2001). MMR deficiency, identified by the presence of microsatellite instability (MSI), occurs in approximately 10–15% of sporadic CRCs (Kinzler and Vogelstein, 1996). Most sporadic CRCs with MSI have been demonstrated to be caused by somatic hypermethylation of the *MLH1* promoter region, resulting in the down-regulation of *MLH1* gene expression (Herman *et al*, 1998). Recent data have revealed that immunohistochemistry is an accurate screening technique to identify MMR deficient tumours (Thibodeau *et al*, 1996; Marcus *et al*, 1999).

Recently, Fong *et al* (2000) have demonstrated that NMBA (N-nitrosomethylbenzylamine) exposure caused a spectrum of visceral and skin tumours similar to Muir-Torre syndrome, caused by a deficiency in a MMR gene, in Fhit-deficient mice, and suggested that the *FHIT* gene may be a target of damage in a fraction of mismatch deficient tumours. Moreover, Mori *et al* (2001) reported that the loss of Msh2 protein is significantly correlated with the loss of Fhit expression in human CRCs. However, the relationship between Fhit and the MMR gene (both Msh2 and Mlh1) expression has not been previously studied in detail using clinical samples.

In this study, we examined the immunohistochemical expression of Fhit, Msh2, Mlh1, and p53 in advanced CRCs to explore the hypothesis that the Fhit inactivation is a frequent result of MMR deficiency.

## MATERIALS AND METHODS

### Patient samples

Tumour blocks were obtained from 52 patients, who underwent colorectomy at Tottori University Hospital between 1992 and 1996. Patients with familial adenomatous polyposis (FAP) were excluded, but family history was not an exclusion criterion; therefore, some cases of HNPCC may have been included in the study population. The ages of the 27 male and 25 female patients ranged from 51 to 90 years (mean±SD: 67.4±1.32 years). Pathologically, all of the tumours were advanced adenocarcinomas (19 well-differentiated, 17 moderately-differentiated, and 16 poorly-differentiated). Nineteen cases had right sided tumours and 33 cases had left sided tumours. Right-sided lesions were defined as those confined to the coecum or ascending or transverse colon, whereas left-sided lesions were defined as being confined to the descending colon, sigmoid colon, or rectum. On the basis of Dukes' classification, four were in stage A, 12 in stage B, 21 in stage C, and 15 in stage D. Lymph node metastases were present in 34 of the 52 (59.6%) patients. Histological classification was made according to the criteria of the Colorectal Cancer Study Group of Japan (Colorectal Cancer Study Group of Japan, 1998). All diagnoses of pathological specimens were verified by two experienced pathologists (H.A. and H.I.). All the cases were analysed anonymously, i.e., all the specimens were given new numbers without names. Institutional Review Board approval was obtained.

### Immunohistochemical staining

Paraffin-embedded, 4 μm-thick sections were immunohistochemically stained with anti-FHIT rabbit polyclonal antibody (IBL, Gunma, Japan; dilution 1 : 100), anti-MSH2 mouse monoclonal antibody (FE11, Oncogene Research Products, Cambridge, MA, USA); dilution 1 : 100), anti-MLH1 mouse monoclonal antibody (G168-15, PharMingen, San Diego, CA, USA; dilution 1 : 50), and anti-p53 mouse monoclonal antibody (DO-7, Dakopatts, Copenhagen, Denmark; dilution 1 : 50) using the avidin-biotin-peroxidase complex technique. Immunohistochemical staining was performed as described below. In brief, after deparaffinising in xylene and dehydrating in ethanol, the sections were immersed in a citrate buffer (0.01 M, pH 6.0) and heated in a microwave oven for 15 min to retrieve antigens, then incubated with the primary antibody overnight at 4°C. As a negative control, the primary antibody was replaced with normal serum IgG in a similar dilution. The detection reaction followed the Vectastain Elite ABC kit protocol (Vector Laboratories, Burlingame, CA, USA). Diaminobenzidine was used as a chromogen, and methylgreen or haematoxylin was used as a counterstain. The sections were then incubated with biotinylated anti-rabbit IgG and avidin-biotin-peroxidase and visualised using diaminobenzidine tetrahydrochloride. The immunohistochemical expressions were evaluated by two independent observers (H.A. and K.Y.). Immunohistochemical analysis was performed in a blinded manner with respect to the clinical information.

### Assessment of Fhit immunostaining

The Fhit expression was graded for both the extent and the intensity of immunopositivity as described previously Hao *et al* (2000). The extent of positivity was scored as follows: 0, <5%; 1, >5–25%; 3, >50–75%; and 4, >75% of the colonic epithelial cells in the respective lesions. The intensity was scored as follows: 0, negative; 1+, weak; 2+, moderate; and 3+, as strong as normal mucosa. The final score was obtained by multiplying the extent of positivity and intensity scores, producing a range from 0 to 12. Scores 9–12 were defined as preserved or strong staining pattern, scores 0–4 were defined as markedly reduced or lost expression, and scores 5–8 were defined as intermediate staining pattern.

### Assessment of Msh2 and Mlh1 immunostaining

Normal tissue adjacent to the tumour was used as an internal positive control. The normal staining pattern for both hMlh1 and hMsh2 was nuclear. Tumour cells that exhibited an absence of nuclear staining in the presence of non-neoplastic cells with nuclear staining were considered to have an abnormal pattern.

### Assessment of p53 immunostaining

Five representative fields were examined, and a total of 1000 tumour cells (200 for each field) were counted under the microscope with a high power (×200) objective. A distinct nuclear immunoreaction was assessed as positive. In this study, the specimens were regarded as p53-positive when over 25% of the tumour cells showed positive signals.

### Statistical evaluation

Statistical analysis was performed by the Fisher's exact test. *P*<0.05 was considered significant.

## RESULTS

### Fhit expression in the normal mucosa and advanced cancers

**Figure 1:**
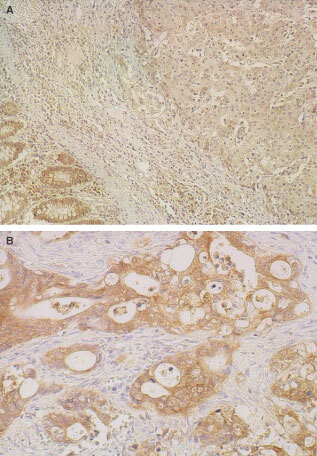
Representative results of Fhit immunostaining in human normal and carcinomatous colorectal tissues. (**A**), Reduced immunostaining of a tumour and positive immunostaining of normal colonic epithelium; (**B**), Positive immunostaining of an invasive tumour

By immunohistochemical staining, all the normal colonic epithelia showed strong cytoplasmic expression of the Fhit protein from the basal cells to the luminal differentiated cells (Figure 1A

); these served as internal controls. Smooth muscle cells and inflammatory mononuclear cells were positive at various intensities and to various degrees.

Reduced or absent Fhit expression was noted in 18 of the 52 (34.6%) advanced CRC cases, preserved in 25 (48.1%) CRCs and intermediate in nine (17.3%) CRCs (Table 1

**Table 1:**
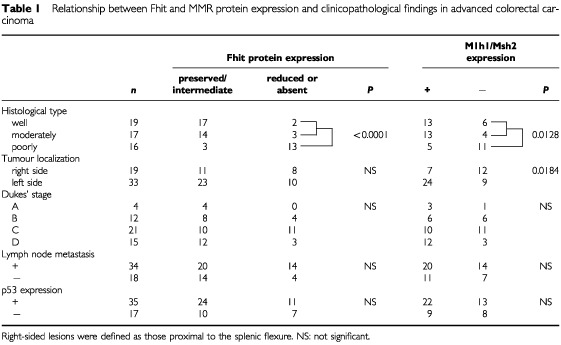
Relationship between Fhit and MMR protein expression and clinicopathological findings in advanced colorectal carcinoma

, Figure 1A,B). The intensity of the staining of the colorectal carcinoma cells was determined by comparing it to that of the normal colonic epithelial cells found within the same specimen (Figure 1A).

We analysed the relationship between these results and the clinicopathological data (tumour location, histological differentiation, Dukes' stage, lymph node metastasis, and p53 expression). Decreased staining or lack of staining for Fhit was detected in 2 of 19 (10.5%) well-differentiated cancers, in three of 17 (17.6%) moderately-differentiated cancers, and in the 13 of 16 (81.3%) poorly-differentiated cancers. The incidence of reduced Fhit expression in CRCs was significantly highest in the poorly-differentiated histology (*P*<0.0001; Table 1). However, no significant associations were found among the Fhit expression and other clinicopathological parameters.

### Correlation of Fhit expression with MMR gene expression

**Figure 2:**
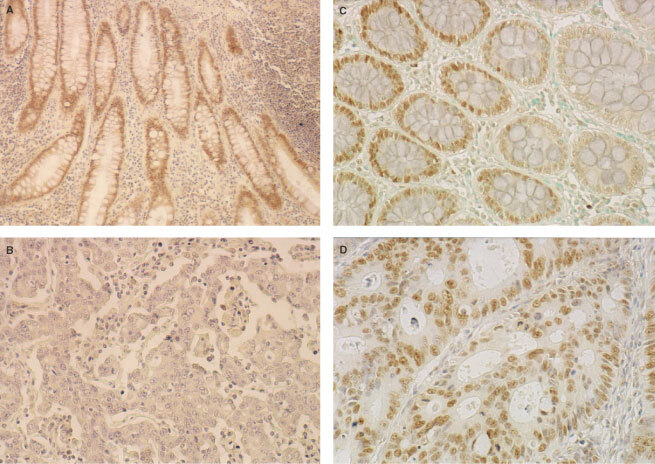
Representative results of Mlh1 and Msh2 immunostaining in human normal and carcinomatous colorectal tissues. **A** and **B**, Poorly-differentiated carcinoma cells (**B**) show no nuclear staining for Mlh1 while normal crypt epithelium (**A**) shows nuclear staining; (**C**) Normal crypt epithlium shows nuclear staining for Msh2; (**D**), Moderately-differentiated carcinoma cells show nuclear staining for Msh2

The expression of both Mlh1 and Msh2 proteins was without exception nuclear. In the normal mucosa, they were detected predominantly in the areas of active proliferation, such as the germinal centres of the lymphoid follicles and the lower portions of the normal colonic crypts (Figure 2A,C

). Normal stromal cells such as fibroblasts and endothelial cells also showed nuclear positivity for both the proteins. We demonstrated that 19 of the 52 (36.5%) tumours had reduced expression levels of the Mlh1 protein (Figure 2B), whereas five (9.6%) carcinomas had reduced expression levels of the Msh2 protein (Figure 2D). Reduced expression levels of both proteins were observed in three of the 52 (5.8%) specimens (Table 2

**Table 2:**
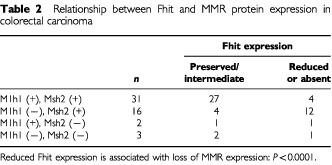
Relationship between Fhit and MMR protein expression in colorectal carcinoma

). In addition, loss of nuclear Mlh1 or Msh2 expression was more frequently associated with poor differentiation (*P*=0.0128) and right-sided location (*P*=0.0184) (Table 1). However, there was no significant difference in other clinicopathological parameters (age, gender, Dukes stage, node metastasis, and p53 expression). Among the tumours with reduced or absent Fhit expression, 72.2% (14 of 18) had loss of nuclear Mlh1 or Msh2 expression compared with only 20.6% (seven of 34) of the preserved or intermediate Fhit expression tumours (*P*<0.0001) (Table 2).

## DISCUSSION

Alterations and abnormal transcripts of the *FHIT* gene have been reported in a number of primary human tumours, including CRCs (Ohta *et al*, 1996; Croce *et al*, 1999; Kitamura *et al*, 2001). However, some of the data are conflicting in CRCs (Hao *et al*, 2000; Hibi *et al*, 1997; Luceri *et al*, 2000; Mori *et al*, 2001; Thiagalingam *et al*, 1996). Recently, it was reported that alterations in the *FHIT* locus detected by DNA and/or reverse transcription-PCR analysis correlated with a loss of Fhit protein expression in lung, cervical, and oesophageal carcinomas (Birrer *et al*, 1999; Mori *et al*, 2000; Sozzi *et al*, 1998). These results indicated that *FHIT* gene alteration can be simply detected by immunohistochemical analysis of tumour specimens. In this study, we observed frequent abnormal Fhit protein expression in poorly-differentiated CRCs, with 77% of the specimens demonstrating a decrease in, or lack of, Fhit protein staining. This frequency of abnormal Fhit expression was similar to that observed in poorly-differentiated CRCs by Hao *et al* (2000). Therefore, these results suggest that abnormal Fhit expression is associated with decreasing degrees of differentiation in CRCs. However, we found no correlation between the Fhit expression and any of the clincopathological parameters including p53 expression.

Inactivation of MMR genes is a recently described alternate pathway in cancer development and progression (Eshleman and Makowitz, 1996; Kinzler and Vogelstein, 1996). MMR deficiency is present in 10–15% of sporadic CRCs (Kinzler and Vogelstein, 1996), and is the underlying cause of more than 90% of HNPCC (Peltomaki and Vasen, 1997). Previous studies demonstrated that immunohistochemical analysis of the expression of Mlh1 and Msh2 could be an accurate and rapid screening procedure for the identification of MMR gene alterations (Thibodeau *et al*, 1996; Marcus *et al*, 1999). We found abnormal Mlh1 or Msh2 expression in 21 of the 52 (46%) CRCs using immunohistochemical methods. This frequency of abnormal MMR protein expression was more frequent than that observed in CRC by other investigators (Kinzler and Vogelstein, 1996). In the assessment of MMR protein expression, we used almost the same criteria used by other groups (Marcus *et al*, 1999; Ward *et al*, 2001). In addition, it was reported that sporadic CRCs with MSI were more frequent in the poorly differentiated phenotype (Ward *et al*, 2001). In our study, the ratio of poorly differentiated CRCs was high compared with previous reports (Kinzler and Vogelstein, 1996; Marcus *et al*, 1999; Ward *et al*, 2001). Therefore, high frequency of abnormal MMR protein expression might be mainly due to the different distributions of histological differentiation. Moreover, we found a significant correlation between the MMR protein expression and histological differentiation and tumour location. These results were also consistent with previous reports (Kinzler and Vogelstein, 1996). In the present study, MMR deficiency was mainly due to the loss of Mlh1 expression, suggesting that *MLH1* hypermethylation is the predominant mechanism (Herman *et al*, 1998).

Recently, it has been reported that by NMBA (N-nitrosomethylbenzylamine) exposure, Fhit-deficient mice developed a spectrum of visceral and skin tumours similar to Muir-Torre syndrome, caused by a deficiency in a MMR gene (Fong *et al*, 2000). A large subgroup of MTS cases exhibits MSI and germline mutations in the *MLH1* or *MSH2* gene (Kruse *et al*, 1998). In addition, it was previously observed that human pancreatic cancers and cell lines with high MSI frequently had homozygous deletions within *FHIT* (Hilgers and Kern, 1999; Hilgers *et al*, 2000). Therefore, these reports suggested that the *FHIT* gene might be a target of damage in a fraction of mismatch deficient tumours. Our data show that MMR deficiency based on the status of the Mlh1 and Mlh2 protein expression is significantly associated with reduced Fhit expression in advanced colorectal carcinomas and support this hypothesis. Mori *et al* (2001) reported an association between Msh2 absence and *FHIT* alterations in CRCs but did not analyse Mlh1 expression. Moreover, as the mechanisms of this hypothesis, they proposed that the repetitive elements, such as (CA)n and (A)n repeats, in introns 4 and 5 of the *FHIT* gene could be a target of damage in MMR deficient tumours.

Thus, we noted an association between Fhit and MMR protein expression, but it could be due to the result of an association between Fhit and MMR protein expression and poorly differentiation.

In conclusion, we have demonstrated that reduced Fhit expression was associated with the loss of MMR protein expression in advanced CRCs. In combination with previous reports, this result supports the possibility that *FHIT* gene alterations could be caused by a deficiency in a MMR gene. Studies that look into the regulation of Fhit and MMR protein expression in cancer may offer a new insight into colorectal carcinogenesis and plausibly, chemopreventive pathways.
